# Efficacy of Ustekinumab combined with partial enteral nutrition in Crohn’s disease

**DOI:** 10.3389/fnut.2026.1825135

**Published:** 2026-05-20

**Authors:** Hanyu Yin, Chao Chen, Xiaowei Chen, Weixiang Yao, Chen Huang, Jiahe Chen, Mengying Zhu, Xiaoli Wu

**Affiliations:** 1 Department of Gastroenterology, The First Affiliated Hospital of Wenzhou Medical University, Wenzhou, Zhejiang, China; 2 Department of Gastroenterology, Xianju County People's Hospital, Xianju, Zhejiang, China

**Keywords:** clinical remission, Crohn’s disease, endoscopic remission, partial enteral nutrition, Ustekinumab

## Abstract

**Background:**

Ustekinumab (UST) has emerged as an effective biological therapy for Crohn’s disease (CD), yet many patients experience suboptimal long-term mucosal healing. Partial enteral nutrition (PEN) may enhance treatment outcomes by reducing intestinal inflammation and improving nutritional status. However, evidence regarding the combined use of UST and PEN remains limited. This study aims to investigate the effect of UST combined with PEN on therapeutic efficacy in CD patients.

**Methods:**

We conducted a single-center, retrospective cohort study including adult CD patients who received UST induction and maintenance therapy. Patients were stratified into a UST + PEN group and a UST monotherapy group according to whether ≥50% of their calculated daily energy intake was provided by PEN. Clinical outcomes (CDAI at weeks 8 and 24) and the primary endpoint endoscopic remission at week 54 (SES-CD < 3 without ulcers) were assessed. Multivariable logistic regression was used to identify independent predictors of endoscopic remission.

**Results:**

A total of 124 patients were included, with 38 receiving UST + PEN. Despite having lower BMI and higher baseline CDAI, the endoscopic remission rate at week 54 was significantly higher in the combination group (71.05% vs. 50.00%, *p* = 0.029). Multivariable analysis demonstrated that combining PEN with UST was an independent protective factor for endoscopic remission [OR 2.84 (95% CI 1.11–7.26), *p* = 0.0289]. Subgroup analyses showed consistent benefit across demographic and disease-related subgroups.

**Conclusion:**

Ustekinumab combined with partial enteral nutrition is associated with higher rates of endoscopic remission at week 54 in patients with Crohn’s disease.

## 1 Introduction

Crohn’s disease (CD) is a chronic, relapsing inflammatory bowel disorder that primarily affects the terminal ileum and adjacent colon (1). Its characteristic clinical features include recurrent abdominal pain, persistent diarrhea, palpable abdominal masses, and perianal lesions. Severe cases may involve complications including intestinal obstruction, fistulas, or abscesses (2). Epidemiological data indicate a significant rise in CD incidence in newly industrialized regions, including parts of Asia, highlighting its growing importance as a global health challenge (3–5). The etiology remains unclear, but it is generally believed to be closely related to genetic susceptibility (e.g., mutations in genes such as NOD2, ATG16L1, IL-23R), environmental factors (such as smoking and unhealthy diet), imbalance of the intestinal microbiota, and abnormal immune responses (1). Studies have shown that the chronic inflammation in CD is mainly driven by overactivation of Th1 and Th17 cells (6). Th1 cells secrete IFN-*γ* to promote cell-mediated immune responses, whereas Th17 cells secrete IL-17/IL-22 to promote neutrophil infiltration and mucosal inflammation. IL-12 and IL-23 are heterodimeric cytokines that share the p40 subunit and are produced by innate immune cells such as macrophages and dendritic cells. They drive Th1 and Th17 differentiation, respectively. These two cytokines are considered core drivers of intestinal inflammatory responses, with IL-23 being especially important in the pathogenesis of small-bowel inflammation in CD (7). Ustekinumab (UST) is a fully human IgG1κ monoclonal antibody that specifically binds the shared p40 subunit of IL-12 and IL-23, thereby blocking their interaction with the IL-12Rβ1 receptor. This dual blockade suppresses both the IL-12-mediated Th1 pathway and the IL-23-mediated Th17 pathway, significantly reducing the production of inflammatory cytokines such as IFN-*γ*, TNF-*α*, and IL-17 (8). Structural and functional studies have shown that after binding p40, UST effectively neutralizes the biological activity of IL-12 and IL-23, thereby inhibiting their pro-inflammatory signaling (9).

UST was initially approved for moderate-to-severe plaque psoriasis (10), and subsequent clinical trials confirmed its significant efficacy in CD and ulcerative colitis. The approved use of UST includes adult patients (aged 18 years or older) diagnosed with CD of moderate-to-severe activity. It is indicated as a treatment option following an insufficient response to or intolerance of conventional or anti-TNF-*α* therapies (11, 12). For example, a 3-year follow-up study found that even in a cohort with a high proportion of prior anti-TNF exposure, UST still achieved meaningful clinical remission and favorable drug persistence (13). The American Gastroenterological Association also recommends UST for patients who previously did not respond to anti-TNF agents. Overall, these data further clarify UST’s role in the current CD treatment algorithm, namely as an important first-line or second-line biologic option, especially for patients with inadequate response to anti-TNF therapy. However, despite these benefits, loss of response still occurs in a subset of patients (14).

Chronic inflammation in CD often leads to impaired intestinal nutrient absorption, increased nutritional requirements, and nutrient loss, resulting in a higher risk of malnutrition in patients. Furthermore, the nutritional status of CD patients correlates strongly with their risk of adverse clinical outcomes (15). Accumulating evidence indicates that nutritional interventions play a key role in influencing both the development and course of Crohn’s disease (CD), representing a valuable adjunct to standard therapeutic regimens (16, 17). Enteral nutrition (EN) involves delivering a liquid diet orally or through a nasogastric tube and is categorized into two types. Exclusive enteral nutrition (EEN) involves the sole use of a nutritional formula to meet all energy requirements, excluding any ordinary solid food. Partial enteral nutrition (PEN), on the other hand, combines formula feeding with the consumption of a portion of the patient’s habitual solid diet (18). Our previous cohort study demonstrated that CD patients treated with infliximab (IFX) combined with PEN had a higher clinical response rate (87.01% vs. 74.75% at week 14, *p* = 0.043) and a higher long-term endoscopic remission rate (84.42% vs. 65.66% at week 54, *p* = 0.005) compared to those receiving IFX alone, and that PEN was an independent protective factor for maintaining endoscopic remission (19). Another retrospective study of adult CD patients who underwent UST induction noted that enteral nutrition (whether EEN or PEN) not only effectively corrected malnutrition but also synergistically alleviated clinical symptoms, reduced intestinal mucosal inflammation, and promoted mucosal healing (20). In children affected by mild-to-moderate CD, research from a prospective randomized controlled trial has shown that integrating PEN with the Crohn’s Disease Exclusion Diet (CDED) presents a more practical and tolerable alternative to traditional EEN. This integrated dietary intervention not only promoted clinical remission through a synergistic action but also resulted in the prolonged suppression of inflammatory biomarkers at both systemic and intestinal levels (21). Collectively, the research suggests a synergistic effect, where nutritional interventions augment the therapeutic impact of biologics used for CD.

No previous research has evaluated the endoscopic results over extended periods when using PEN alongside UST in a systematic manner. We therefore conducted this retrospective cohort study to determine if this integrated approach can concurrently improve nutritional status and disease activity in CD, with the ultimate objective of elevating clinical and endoscopic remission rates.

## 2 Materials and methods

### 2.1 Patient Enrollment

Patients were consecutively recruited from January 2021 to November 2025 at the First Affiliated Hospital of Wenzhou Medical University. All participants fulfilled the predefined eligibility criteria: (1) A confirmed CD diagnosis per the Chinese Crohn’s Disease Diagnosis and Treatment Guidelines (2023, Guangzhou); (2) Receiving UST induction and maintenance therapy; (3) Complete baseline information and continued regular UST therapy through the week 54 endoscopic examination. The exclusion criteria encompassed: (1) Incomplete baseline data or failure to follow up continuously for 1 year or loss to follow-up; (2) Patients receiving EEN or those not meeting the definition of PEN; (3) Presence of comorbid conditions known to confound laboratory assessments, including cirrhosis, malignancies, autoimmune disorders, or severe cardiovascular/cerebrovascular diseases.

### 2.2 UST induction and maintenance therapy

UST induction was administered as a single intravenous infusion at week 0, using the approved weight-based dosing algorithm of 260 mg for patients weighing <55 kg, 390 mg for those between 55 and 85 kg, and 520 mg for individuals weighing >85 kg. After induction, all patients transitioned to subcutaneous maintenance therapy with UST 90 mg every 8 weeks. Throughout the 54-week observation period, no dose escalation or interval shortening was performed, and all patients remained on the standard q8w regimen without any treatment intensification.

### 2.3 PEN protocol and grouping

The daily energy requirements for patients with CD were assessed by a clinical dietitian and estimated at 30–35 kcal per kilogram of body weight. PEN was administered orally using either polymeric (e.g., Fresubin Energy) or short-peptide (e.g., Peptisorb) formulas.

Patient allocation followed strict, mutually exclusive criteria to maintain a clear distinction between treatment groups. Patients were assigned to the UST + PEN group only if they consistently met at least 50% of their daily energy needs through PEN over the full 54-week treatment period, as confirmed by regular nutritional assessments. In contrast, those who never received PEN during the 54-week period were assigned to the UST monotherapy group. Furthermore, individuals with intermittent, short-term, or inconsistent use of enteral nutrition that fell short of the sustained 50% energy threshold were excluded from the UST + PEN group.

### 2.4 Data collection and treatment evaluation

Baseline demographics and clinical characteristics were obtained from past medical records. These included standard demographic factors (age, gender, BMI), behavioral histories (smoking, alcohol), disease-specific details (duration, Montreal classification), and treatment history (prior CD surgeries, previous biologic agent use). Data compilation also included disease activity markers under UST therapy. Laboratory values (CRP, ALB, ESR) and CDAI scores were obtained at baseline, week 8, and week 24. Furthermore, we recorded the endoscopic findings at week 54, along with the number of times enteral nutrition was administered. For the assessment of the primary endpoint, endoscopic remission at week 54, ileocolonoscopy images were independently reviewed by two experienced gastroenterologists who were blinded to treatment allocation and clinical outcomes. Each reviewer assigned SES-CD segmental subscores (ulcer size, ulcerated surface, affected surface, and narrowing), which were summed to generate the total SES-CD score. In cases of discrepancy between reviewers, the final score was determined through consensus discussion prior to statistical analysis.

In addition to routine laboratory tests, we calculated the C-reactive protein-to-albumin ratio (CAR) by dividing the serum CRP (mg/L) by ALB (g/L) to obtain a composite marker of systemic inflammation and nutritional status. Based on prior reports, patients were stratified into low (<0.05) and high (≥0.05) CAR groups for analysis, using 0.05 as the threshold value.

Adherence to PEN was assessed based on available medical records, dietary documentation, and routine clinical follow-up notes. Due to the retrospective nature of the study, precise quantification of adherence could not be fully ensured.

### 2.5 Statistical analysis

Continuous variables were reported as mean ± standard deviation (SD) or median (interquartile range, IQR). For comparisons between groups, the Student’s *t*-test and the Mann–Whitney U test were employed for normally distributed and non-parametric data, respectively. Intra-group comparisons were performed using the Wilcoxon signed-rank test. Categorical variables are summarized as frequencies (percentages), and group differences were tested with the Chi-square or Fisher’s exact test, as appropriate. Univariate logistic regression yielded ORs and 95% CIs for the association of baseline traits with endoscopic remission. Determinants of week 54 remission were identified via multivariate logistic regression, incorporating variables significant at *p* < 0.10 in univariate analysis or those altering the PEN-therapy coefficient by >10%. Effect modification was assessed through stratified subgroup analyses (age, sex, BMI, disease location, behavior, surgery history), with interaction terms tested via likelihood ratio tests. Analytical procedures were executed using SPSS 26.0 (SPSS, Chicago, Illinois), EmpowerStats, and R. Statistical significance for all two-sided tests was defined as *p* < 0.05.

To reduce potential time-dependent bias, patients were required to have completed at least 54 weeks of follow-up in both groups. Missing data were handled using a complete-case analysis approach. Patients with incomplete baseline data, loss to follow-up, or missing week 54 endoscopic assessment were excluded according to the predefined exclusion criteria. For the remaining included cohort, the proportion of missing values for laboratory parameters was low (<5%), and no additional imputation procedures were performed. Sensitivity analyses excluding variables with minor missingness yielded consistent results.

## 3 Results

### 3.1 Patient baseline characteristics

**Figure 1:**
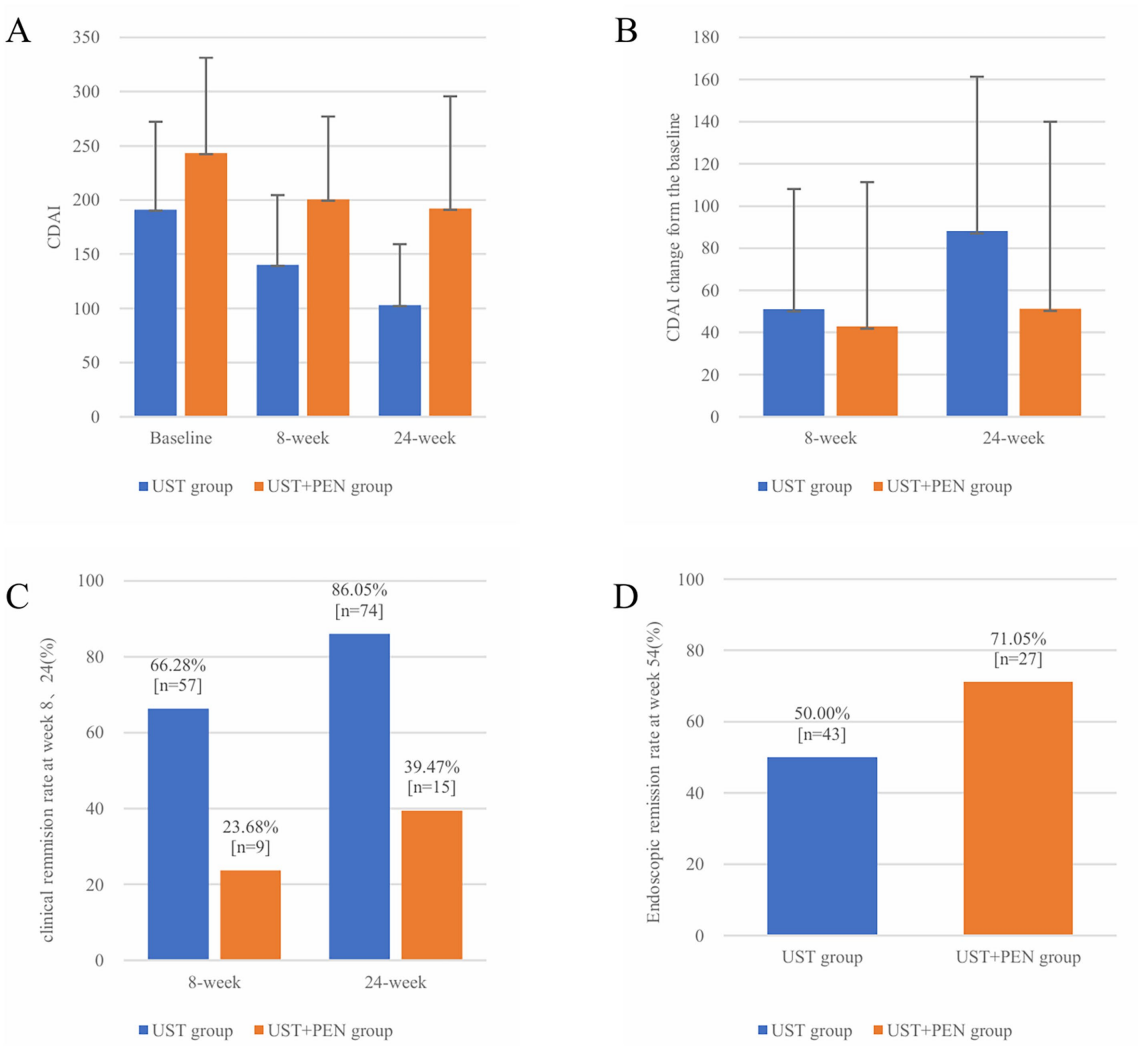
Efficacy between the UST monotherapy group and UST + PEN group. **(A)** CDAI scores at baseline, week 8, and week 24 in two groups. **(B)** CDAI score change during 8-week and 24-week period in two groups. **(C)** The number and proportion of CD patients achieving clinical remission at week 8 and week 24. **(D)** The number and proportion of CD patients achieving endoscopic remission at week 54.

A total of 124 patients with CD were included in the analysis, of whom 86 (69.4%) received UST monotherapy and 38 (30.6%) received UST combined with PEN. As shown in Table 1 and Figure 1, several baseline characteristics differed significantly between groups. Specifically, the UST + PEN group had a lower mean BMI (18.19 ± 2.23 vs. 20.56 ± 3.03 kg/m^2^, *p* < 0.001) and higher CDAI scores (243.14 ± 88.03 vs. 191.07 ± 81.10, *p* = 0.002) than those receiving UST monotherapy. The UST + PEN group comprised more male patients and a greater frequency of penetrating disease. Prior biologic exposure differed significantly between groups (*p* < 0.05). No significant differences were observed in CRP, ESR, ALB, or Montreal classification categories.

**Table 1:** Baseline characteristics of study cohorts.

Variable	Total (*n* = 124)	UST group (*n* = 86)	UST + PEN group (*n* = 38)	*p*-value
Age (years)	35.21 ± 12.17	34.10 ± 12.07	37.71 ± 12.19	0.129
Disease duration (months)	24.00 (6.00–75.00)	24.00 (4.25–72.00)	24.00 (9.00–84.00)	0.733
BMI (kg/m^2^)	19.83 ± 3.01	20.56 ± 3.03	18.19 ± 2.23	<0.001
CDAI score	207.08 ± 86.40	191.07 ± 81.10	243.14 ± 88.03	0.002
SES-CD	9.50 (6.00–13.00)	10.00 (6.00–14.75)	8.00 (6.00–11.00)	0.137
CRP (mg/L)	9.15 (3.35–21.72)	9.65 (4.70–21.52)	6.70 (2.02–20.28)	0.691
ALB (g/L)	38.56 ± 4.64	39.02 ± 4.36	37.52 ± 5.12	0.096
ESR (mm/h)	10.00 (4.75–21.00)	11.00 (5.00–20.75)	8.00 (4.00–22.00)	0.549
Sex				0.029
Female, *n* (%)	30 (24.19)	16 (18.60)	14 (36.84)	
Male, *n* (%)	94 (75.81)	70 (81.40)	24 (63.16)	
Age at diagnosis, *n* (%)				0.490
A1 (≤16 years), *n* (%)	0 (0.00)	0 (0.00)	0 (0.00)	
A2 (17–40 years), *n* (%)	90 (72.58)	64 (74.42)	26 (68.42)	
A3 (>40 years), *n* (%)	34 (27.42)	22 (25.58)	12 (31.58)	
Disease location				0.146
L1 (ileal), *n* (%)	34 (27.42)	19 (22.09)	15 (39.47)	
L2 (colonic), *n* (%)	10 (8.06)	6 (6.98)	4 (10.53)	
L3 (ileocolonic), *n* (%)	79 (63.71)	60 (69.77)	19 (50.00)	
L4 (upper digestive tract involved), *n* (%)	19 (15.32)	12 (13.95)	7 (18.42)	0.524
Disease behavior				0.046
Structuring and penetrating, *n* (%)	12 (9.68)	9 (10.47)	3 (7.89)	
B1 (non-structuring and penetrating), *n* (%)	54 (43.55)	42 (48.84)	12 (31.58)	
B2 (structuring), *n* (%)	63 (50.81)	42 (48.84)	21 (55.26)	
B3 (penetrating), *n* (%)	19 (15.32)	11 (12.79)	8 (21.05)	
Perianal disease, *n* (%)	75 (60.48)	50 (58.14)	25 (65.79)	0.422
Extra-intestinal manifestations, *n* (%)	5 (4.03)	4 (4.65)	1 (2.63)	0.598
Smoking status, *n* (%)	10 (8.06)	6 (6.98)	4 (10.53)	0.503
CD-related surgical history, *n* (%)	66 (53.23)	42 (48.84)	24 (63.16)	0.141
Concomitant immunomodulatory treatment, *n* (%)	24 (19.35)	14 (16.28)	10 (26.32)	0.192
Use of biologics for the first time, *n* (%)	84 (67.74)	64 (74.42)	20 (52.63)	0.017

UST, ustekinumab; PEN, partial enteral nutrition; CD, Crohn’s disease; BMI, body mass index; CDAI, Crohn’s disease activity index; SES-CD, simple endoscopic score for Crohn’s disease; CRP, C-reactive protein; ALB, albumin; ESR, erythrocyte sedimentation rate.

These baseline differences indicate that patients in the UST + PEN group had a more severe disease profile at enrollment, which may have influenced early treatment response.

### 3.2 Efficacy assessment at weeks 8, 24, and 54

According to the data shown in Table 2, the UST + PEN group began with a marked elevation in CDAI score compared to the UST monotherapy group (243.14 ± 88.03 vs. 191.07 ± 81.10, *p* = 0.002), and this difference persisted at weeks 8 and 24 (both *p* < 0.001). Correspondingly, the clinical remission rates at weeks 8 and 24 were significantly higher in the UST group than in the combination group (week 8: 66.28% vs. 23.68%; week 24: 86.05% vs. 39.47%; both *p* < 0.001). However, at the week 54 endoscopic evaluation, the endoscopic remission rate demonstrated a clear advantage for the UST + PEN group compared to the UST monotherapy group (71.05% vs. 50.00%, *p* = 0.029). These early differences may partly reflect the more severe baseline disease activity and poorer nutritional status in the UST + PEN group. Importantly, despite this less favorable starting profile, the combination group still achieved a higher week 54 endoscopic remission rate. In terms of laboratory indicators, there were no significant differences in CRP or ESR between the two groups at week 24.

**Table 2:** CDAI scores and efficacy evaluation at week 8, week 24, and week 54 between the UST group and the UST + PEN group.

Variable	UST group (*n* = 86)	UST + PEN group (*n* = 38)	*p*-value
CDAI score at baseline	191.07 ± 81.10	243.14 ± 88.03	0.002
CDAI score at week 8	140.06 ± 64.42	200.33 ± 76.73	<0.001
Clinical remission at week 8, n (%)	57 (66.28)	9 (23.68)	<0.001
CDAI score at week 24	103.09 ± 56.24	191.91 ± 103.85	<0.001
Clinical remission at week 24, n (%)	74 (86.05)	15 (39.47)	<0.001
CRP at week 24	5.60 ± 9.34	9.09 ± 15.94	0.130
ESR at week 24	6.50 ± 7.76	8.03 ± 9.94	0.357
Endoscopic remission at week 54, n (%)	43 (50.00)	27 (71.05)	0.029

UST, ustekinumab; PEN, partial enteral nutrition; CDAI, Crohn’s disease activity index; BMI, body mass index; CRP, C-reactive protein; ESR, erythrocyte sedimentation rate.

### 3.3 Predictors of endoscopic remission

As shown in Table 3, in the follow-up data analysis of included patients, univariate analysis showed that ileal disease (L1) was significantly associated with a higher likelihood of endoscopic remission [OR 5.33 (95% CI 2.01–14.13), *p* = 0.0008], whereas ileocolonic disease (L3) was associated with a lower likelihood of remission [OR 0.32 (95% CI 0.14–0.71), *p* = 0.0050]. Complex structuring or penetrating disease behavior were also negative predictors [OR 0.22 (95% CI 0.06–0.87), *p* = 0.0310]. The UST + PEN treatment regimen itself was a significant predictor of endoscopic remission in the univariate model [OR 2.45 (95% CI 1.08–5.57), *p* = 0.0316]. Multivariable analysis, accounting for age, gender, BMI, disease location, disease behavior, and CD-related surgical history, demonstrated an even stronger and independent protective effect for combined PEN therapy [OR 2.84 (95% CI 1.11–7.26), *p* = 0.0289]. This result confirms the therapy as a significant predictor of achieving endoscopic remission by week 54. Furthermore, in the multivariate model, ileal disease (L1) maintained a significant positive predictive effect [OR 5.74 (95% CI 2.07–15.92), *p* = 0.0008], while ileocolonic disease (L3) retained its negative predictive association [OR 0.32 (95% CI 0.14–0.73), *p* = 0.0071].

**Table 3:** Univariate and multivariable logistic regression analyses for the effect of combination therapy with PEN on endoscopic remission at 54 weeks.

Variable	Non-adjusted model		Multivariate-adjusted Model	
	OR (95% CI)	*p*-value	OR (95% CI)	*p*-value
Age	1.00 (0.97, 1.03)	0.7802	0.99 (0.96, 1.02)	0.4371
Sex				
Female	1.00		1.00	
Male	0.83 (0.36, 1.90)	0.6528	0.90 (0.37, 2.16)	0.8106
BMI	0.98 (0.87, 1.10)	0.6855	1.01 (0.84, 1.21)	0.9232
Disease location				
L1 (ileal)	5.33 (2.01, 14.13)	0.0008	5.74 (2.07, 15.92)	0.0008
L2 (colonic)	0.48 (0.13, 1.81)	0.2819	0.43 (0.11, 1.71)	0.2309
L3 (ileocolonic)	0.32 (0.14, 0.71)	0.0050	0.32 (0.14, 0.73)	0.0071
Behavior				
Structuring and penetrating	0.22 (0.06, 0.87)	0.0310	0.16 (0.04, 0.70)	0.0144
B1 (non-structuring and penetrating)	0.82 (0.40, 1.68)	0.5880	0.94 (0.44, 1.98)	0.8636
B2 (structuring)	1.06 (0.52, 2.15)	0.8746	0.99 (0.48, 2.06)	0.9786
B3 (penetrating)	0.50 (0.19, 1.36)	0.1756	0.33 (0.11, 1.01)	0.0523
CD-related surgical history	0.57 (0.28, 1.17)	0.1235	0.71 (0.30, 1.67)	0.4268
UST	1.00		1.00	
UST + PEN	2.45 (1.08, 5.57)	0.0316	2.84 (1.11, 7.26)	0.0289

BMI, body mass index; CD, Crohn’s disease; UST, Ustekinumab; PEN, partial enteral nutrition. Non-adjusted model adjust for: None. Multivariate-Adjusted Model adjust for: Age; Sex; BMI. Multivariate-Adjusted Model adjust for: Age; Sex; BMI; Disease location; Behavior; CD-related surgical history.

### 3.4 Subgroup and interaction analysis

**Figure 2:**
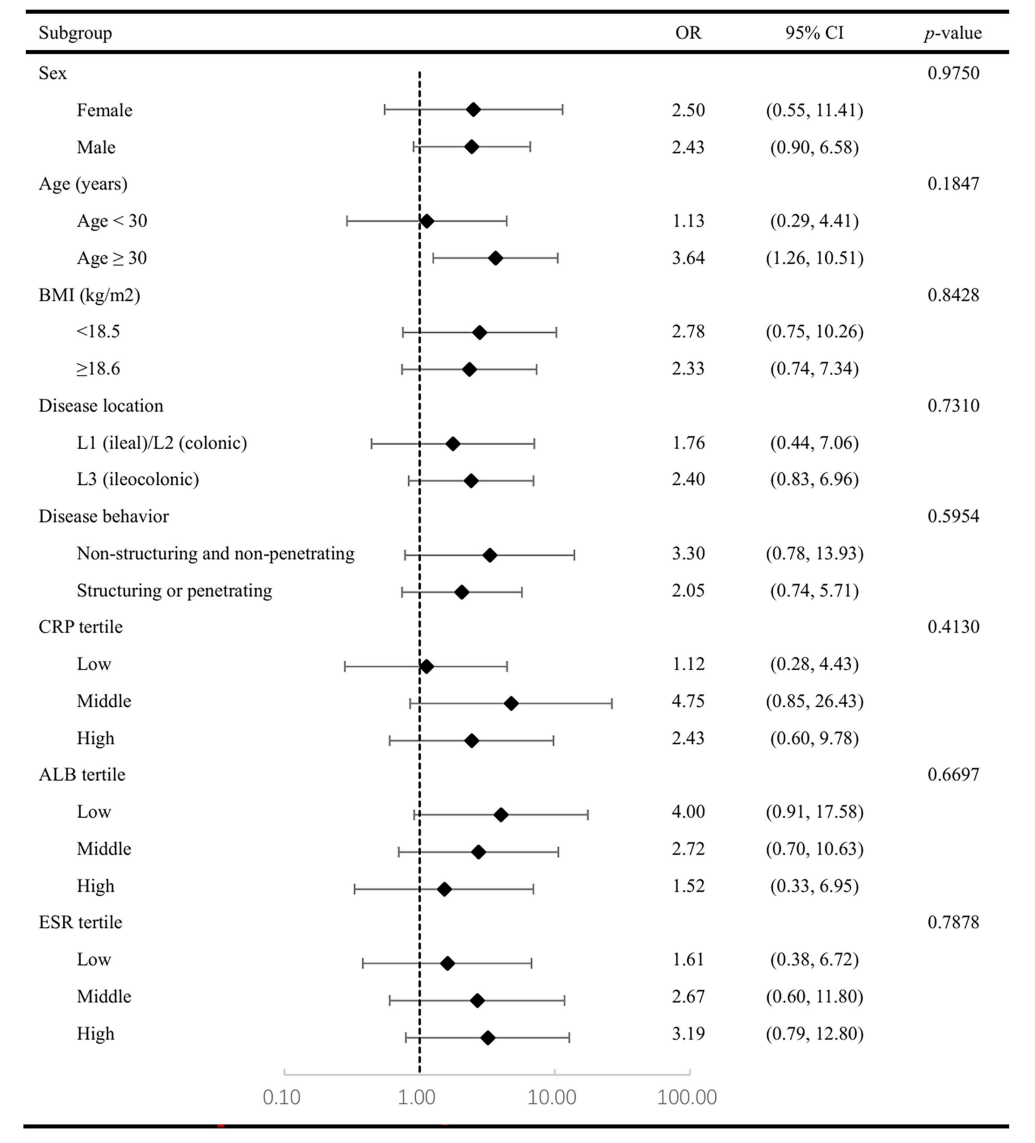
The protective role of combined UST + PEN treatment in achieving endoscopic remission in patients with CD.

The analysis further evaluated endoscopic remission rates with PEN treatment, with detailed stratification according to key demographic and clinical characteristics, including gender, age, BMI, disease location, behavior, and levels of CRP, ALB, and ESR as shown in Figure 2. The results showed that UST combined with PEN exhibited a positive trend toward higher endoscopic remission rates across all strata of baseline characteristics (all subgroup ORs > 1), and the benefit of the combined therapy was statistically significant in patients aged ≥30 years [OR 3.64 (95% CI 1.26–10.51), *p* = 0.0169]. Moreover, the *p*-values for interaction were all >0.05 across subgroups, indicating that the promoting effect of UST + PEN treatment on endoscopic remission was relatively consistent among patients with different gender, age, disease characteristics, and levels of inflammation, and no significant interaction effects with these factors were detected (all interaction *p* > 0.05). The detailed results are summarized in Table 4.

**Table 4:** Association between combination therapy with PEN and CD activity at week 54 according to baseline characteristics.

Variable	UST		UST + PEN		Effect of UST + PEN		*p*-value for interaction
	*N*	Remission, *n* (%)	*N*	Remission, *n* (%)	OR (95% CI)	*p*-value	
Sex							
Female	16	8 (18.60)	14	10 (37.04)	2.50 (0.55, 11.41)	0.2368	0.9750
Male	70	35 (81.40)	24	17 (62.96)	2.43 (0.90, 6.58)	0.0811	
Age (years)							
Age < 30	35	18 (41.86)	11	6 (22.2)	1.13 (0.29, 4.41)	0.8568	0.1847
Age ≥ 30	51	25 (58.14)	27	21 (77.78)	3.64 (1.26, 10.51)	0.0169	
BMI (kg/m^2^)							
<18.5	19	9 (20.93)	21	15 (55.56)	2.78 (0.75, 10.26)	0.1254	0.8428
≥18.6	67	34 (79.07)	17	12 (44.44)	2.33 (0.74, 7.34)	0.1488	
Disease location							
L1 (ileal)/L2 (colonic)	25	17 (39.53)	19	15 (55.56)	1.76 (0.44, 7.06)	0.4221	0.7310
L3 (ileocolonic)	60	25 (58.14)	19	12 (44.44)	2.40 (0.83, 6.96)	0.1068	
Disease behavior							
Non-structuring and non- penetrating	42	20 (46.51)	12	9 (33.33)	3.30 (0.78, 13.93)	0.1042	0.5954
Structuring or penetrating	44	23 (53.49)	26	18 (66.67)	2.05 (0.74, 5.71)	0.1672	
CRP tertile							
Low	22	15 (34.88)	17	12 (44.44)	1.12 (0.28, 4.43)	0.8718	0.4130
Middle	33	14 (32.56)	9	7 (25.93)	4.75 (0.85, 26.43)	0.0752	
High	31	14 (32.56)	12	8 (29.63)	2.43 (0.60, 9.78)	0.2119	
ALB tertile							
Low	26	13 (30.23)	15	12 (44.44)	4.00 (0.91, 17.58)	0.0665	0.6697
Middle	27	10 (23.26)	13	8 (29.63)	2.72 (0.70, 10.63)	0.1503	
High	33	20 (46.51)	10	7 (25.93)	1.52 (0.33, 6.95)	0.5917	
ESR tertile							
Low	23	14 (32.56)	14	10 (37.04)	1.61 (0.38, 6.72)	0.5156	0.7878
Middle	34	17 (39.53)	11	8 (29.63)	2.67 (0.60, 11.80)	0.1962	
High	29	12 (27.91)	13	9 (33.33)	3.19 (0.79, 12.80)	0.1022	

UST, ustekinumab; PEN, partial enteral nutrition; BMI, body mass index; CRP, C-reactive protein; ALB, albumin; ESR, erythrocyte Sedimentation rate.

### 3.5 CAR category changes at week 8

As shown in Table 5, baseline CAR category distribution was comparable between the two groups. At week 8, the distribution of CAR categories differed significantly. In the UST monotherapy group, 22 of 86 patients (25.58%) achieved a CAR < 0.05, compared with 19 of 38 patients (50.00%) in the UST + PEN group. Conversely, CAR ≥ 0.05 was more frequently observed among patients receiving UST alone than in those receiving combination therapy (74.42% vs. 50.00%, *p* = 0.008).

**Table 5:** Comparison of CAR distribution at baseline and week 8 between the UST group and the UST + PEN group.

Variable	UST, *n* (%)	UST + PEN, *n* (%)	*p*-value
Baseline			
CAR < 0.05	11 (12.79)	10 (26.32)	0.064
CAR ≥ 0.05	75 (87.21)	28 (73.68)	
8 week			
CAR < 0.05	22 (25.58)	19 (50.00)	0.008
CAR ≥ 0.05	64 (74.42)	19 (50.00)	

UST, ustekinumab; PEN, partial enteral nutrition; CAR, C-reactive protein-to-albumin ratio.

## 4 Discussion

Ustekinumab (UST), a monoclonal antibody targeting the IL-12/23 p40 subunit, has become one of the relatively safe and effective treatment options for patients with moderate to severe CD (22). However, its integration into routine clinical practice is not without difficulties. According to a meta-analysis conducted by Yang et al., the annual incidence of loss of response in UST-naïve patients is approximately 21%, while the rate of required dose intensification reaches about 25% per patient-year (14). This issue of primary and secondary non-response has prompted interest in combination strategies that may optimize treatment outcomes with UST. Mirroring the paradigm where combining anti-TNF-*α* agents with immunomodulators has proven successful for enhancing therapeutic outcomes (23), pairing UST with other modalities, especially nutritional support such as PEN, represents a potential strategy to improve CD care (24). This study sought to evaluate whether combining UST with PEN was associated with improved outcomes in CD, and found that: (1) Adjunctive PEN therapy during UST maintenance was associated with higher rates of endoscopic remission at week 54. (2) The observed association between combined PEN treatment and endoscopic remission was relatively consistent across patients of different gender, age, disease characteristics, and inflammation levels. (3) The use of combined PEN therapy is independently associated with higher odds of maintaining endoscopic remission in patients with CD.

Huang et al. (19) conducted a retrospective study including 176 CD patients receiving infliximab (IFX). Our results showed that the clinical remission rate at week 14 in CD patients receiving IFX combined with PEN (87.01%) was significantly higher than in the IFX alone group (74.75%), and the endoscopic remission rate at week 54 was also higher (84.42% vs. 65.66%, *p* = 0.005). Another prospective study found that the group receiving adalimumab (ADA) combined with enteral nutrition had better endoscopic and clinical remission rates compared to ADA monotherapy, and both PEN and EEN showed similar efficacy in inducing CD remission (25). In this study, although the UST + PEN group exhibited higher disease activity (CDAI score) and poorer nutritional status (BMI) at baseline, and had significantly lower short-term clinical remission rates at weeks 8 and 24 compared to the UST monotherapy group, the endoscopic remission rate at week 54 was higher than that in the UST monotherapy group (71.05% vs. 50.00%, *p* = 0.029).

A potential explanation for the divergence of early clinical results from later endoscopic remission rates may lie in the poorer baseline nutritional status and higher disease burden in the UST + PEN group. Malnutrition can lead to reduced protein synthesis, decreased immune and reparative capacity, and is closely associated with poor prognosis, increased complications, and higher postoperative recurrence risk (20, 26). Esaki et al. (27) found that one positive predictor of response to UST treatment was a higher serum albumin level (≥3.1 g/dL), whereas elevated baseline CRP (≥1.19 mg/dL) was associated with a lower induction remission rate. These findings may suggest that patients with malnutrition and uncontrolled inflammation could have a less favorable response to UST induction therapy, and although PEN itself may provide supportive benefits, its early advantages are difficult to manifest in patients with more severe disease. Yamamoto et al. (28) conducted a 56-week prospective study which showed that EN combined with IFX during maintenance did not significantly improve the rate of maintaining clinical remission in CD patients (78% in the EN group vs. 67% in the non-EN group, *p* = 0.51), which is consistent with our findings. In addition, previous studies have suggested that EEN may improve nutritional status and modulate inflammatory activity, which could contribute to mucosal recovery, but such improvements usually require several weeks to months before becoming detectable on endoscopy (29, 30). These observations may indicate that PEN was less strongly associated with short-term CDAI improvement, while a stronger association was observed with later mucosal healing outcomes. Malnutrition and systemic inflammation form a bidirectional cycle in CD, in which chronic inflammation increases metabolic demands and protein loss, while inadequate nutritional intake further impairs mucosal healing and immune regulation. Sustained provision of ≥50% of estimated energy requirements through PEN may help attenuate this cycle and potentially create a more favorable condition for biologic therapy response, although this was not directly assessed. It is also noteworthy that, despite higher CDAI scores in the UST + PEN group at weeks 8 and 24, a higher proportion of patients in the UST + PEN group shifted into the low CAR category at week 8, suggesting a more favorable early inflammatory-nutritional profile. This apparent discrepancy likely reflects the different dimensions captured by these indices, as CAR primarily reflects systemic inflammation and nutritional status, whereas CDAI incorporates subjective symptoms and functional impairment that may lag behind biochemical improvement. Although CAR is a non-specific composite marker and causal inference cannot be established in this retrospective design, the observed early shift toward a lower CAR category in the UST + PEN group may reflect an earlier attenuation of systemic inflammation accompanied by nutritional stabilization.

By employing endoscopic remission (defined as SES-CD score <3 and absence of ulcers) as the primary study endpoint, this investigation may strengthen the clinical relevance of the findings. This endpoint aligns with long-term therapeutic goals in CD (31) and reduces reliance on clinical scores, thereby mitigating their inherent subjective bias. The robustness of these findings is further suggested by a multivariable logistic regression analysis that adjusted for key confounders, including age, gender, BMI, disease location, behavior, and CD-related surgical history. This analysis identified the UST + PEN regimen as independently associated with higher odds of endoscopic remission at week 54 [OR 2.84 (95% CI 1.11–7.26), *p* = 0.0289]. The consistency of this association was further explored through subgroup analyses, which showed a generally consistent association across multiple patient subgroups. Although these findings are encouraging, they should be interpreted cautiously given the baseline imbalance and moderate sample size.

Nevertheless, several limitations of this study should be acknowledged. Its single-center retrospective design introduces risks of selection bias, information bias, and incomplete assessment of long-term PEN adherence. The higher baseline disease activity observed in the combination group may reflect underlying treatment selection bias in real-world clinical practice. While statistical adjustments were made, residual confounding remains a possibility. The study’s statistical power is constrained by the moderate overall sample size, particularly the small subgroup (*n* = 38) receiving UST + PEN. As UST is a relatively newer biologic agent in our center, the number of eligible patients treated during the study period remained limited. Variability in adherence among patients may also have contributed to heterogeneity in treatment response. Therefore, large-scale, multicenter, prospective studies with standardized monitoring of nutritional adherence are necessary to confirm the potential clinical value of combining UST with PEN and to define the optimal nutritional support protocol. Future work should also explore how this combined therapy may relate to immune biomarkers and the gut microbiota, insights that will be vital for developing personalized treatment strategies for CD.

## 5 Conclusion

Based on our findings, PEN combination therapy emerged as an independent protective factor for achieving endoscopic remission at week 54 in patients with CD. This supports the view that combining UST with PEN is beneficial for improving endoscopic outcomes.

## Data Availability

The raw data supporting the conclusions of this article will be made available by the authors, without undue reservation.

## Author’s note

The authors confirm that the manuscript (including all data and figures) has not been published previously and is not under consideration for publication elsewhere.
